# Effects of Storage Time and Temperature on Antioxidants in Juice from *Momordica charantia* L. and *Momordica charantia* L. var. *abbreviata* Ser.

**DOI:** 10.3390/molecules25163614

**Published:** 2020-08-09

**Authors:** Yung-Sheng Lin, Wen-Ying Huang, Pang-Yen Ho, Shiou-Yih Hu, Ying-Yi Lin, Cheng-You Chen, Min-Yun Chang, Shu-Ling Huang

**Affiliations:** 1 Department of Chemical Engineering, National United University, Miaoli 36063, Taiwan; linys@nuu.edu.tw (Y.-S.L.); pyho@nuu.edu.tw (P.-Y.H.); everyman2220@gmail.com (S.-Y.H.); a0979169597@gmail.com (Y.-Y.L.); mino101451@gmail.com (M.-Y.C.); 2 Ph.D. Program in Materials and Chemical Engineering, National United University, Miaoli 36063, Taiwan; wayne20410@gmail.com; 3 Institute of Food Safety and Health Risk Assessment, National Yang-Ming University, Taipei 11221, Taiwan; 4 Department of Applied Cosmetology, HungKuang University, Taichung 43302, Taiwan; beca690420@hk.edu.tw

**Keywords:** *Momordica charantia* L., *Momordica charantia* L. var. *abbreviata* Ser., liquid chromatography–mass spectrometry, antioxidant, storage

## Abstract

This study determined the antioxidant activities of juice from *Momordica charantia* L. (MC) and MC var. *abbreviata* Ser. (MCVAS) by analyzing 1,1-diphenyl-2-picrylhydrazyl (DPPH) scavenging ability, ferric reducing power (FRP), and total phenolic content (TPC). The effects of storage time and storage temperature on these antioxidant activities were investigated. Liquid chromatography–mass spectrometry was conducted to identify the major components of MC and MCVAS. The results revealed that the antioxidant activity of MCVAS was better than that of MC, possibly because of richer components of MCVAS. For MC and MCVAS, the scavenging concentrations of 50% DPPH were 3.33 and 1.19 mg/mL, respectively; moreover, the FRP values were 68.93 and 118.14 mg ascorbic acid equivalent/g dry weight, respectively; and the TPC values were 8.15 and 11.47 mg gallic acid equivalent/g dry weight, respectively. The antioxidant activities of MC and MCVAS decreased with storage time. High storage temperature decreased antioxidant activity more quickly than a low temperature. In addition, MC had exhibited a faster decline in DPPH scavenging ability and FRP than MCVAS during 24-day storage, but no difference was observed in TPC.

## 1. Introduction

*Momordica charantia* L. (MC), known as bitter gourd, bitter melon, balsam pear, bitter cucumber, and karela, is a member of the Cucurbitaceae family [1]. MC has diverse characteristics, such as sex expression, growth habits, and maturity, as well as fruit shape, size, color, and surface texture [2]. Medicinal properties of MC that have been studied include antiulcerogenic, antimutagenic, antioxidant, antitumor, antilipolytic, anti-inflammatory, anticarcinogenic, analgesic, abortifacient, antiviral, hypoglycemic, and immunomodulatory properties [3,4,5,6]. MC benefits the circulatory, respiratory, digestive, and nervous systems [7]. Moreover, MC is an excellent source of phenolic compounds and has been applied in dietary supplements [8].

MC var. *abbreviata* Ser. (MCVAS) is a wild species of MC, and it is normally smaller than MC [9]. MCVAS is native to several tropical areas of Asia, including Southern Taiwan [10]. It is widely used as a healthy food and as traditional medicine [11]. MCVAS has various properties and has produced anti-inflammatory, antitumor, antioxidant, and hypoglycemic effects in animal models [10]. MCVAS extracts activated peroxisome proliferator-activated receptor α [9]. Moreover, MCVAS has been reported to be more effective in treating diabetes than MC [11,12].

The bitterness of bitter gourds results from triterpene glycosides (momordicoside K and L) and cucurbitacin-like alkaloids (momordicines I and II) [12,13]. Generally, immature bitter gourd with darker green skin is more bitter, and those with lighter skin tend to be less bitter [8,13,14]. MCVAS is considerably more bitter than MC, possibly because MC contains significantly lower saponin (0.25%) than MCVAS (0.67%) [12,15]. In addition, the beneficial effects of bitter gourd may result from chemical constituents such as cucurbitane-type triterpenoids, cucurbitane-type triterpene glycoside, phenolic acids, flavonoids, essential oils, fatty acids, amino acids, sterols, saponin constituents, and proteins [1]. The pulp of the fruit has higher antioxygenic activity than the seeds, which may be attributed to the presence of different phenolic acids and flavonoids [6,16].

Antioxidant screening of MC has revealed that its extracts exhibit antioxidant effects in vitro [17,18]. A study noted that the 1,1-diphenyl-2-picrylhydrazyl (DPPH) scavenging activity was 17.4 ± 4.55% and 28.2 ± 1.90% in MC extracted with water and ethanol, respectively [19]. The scavenging concentration of 50% DPPH (SC_50_) value for DPPH scavenging activity was 0.2 mg/mL for MC fruit extracted using methanol [20]. Wu and Ng (2008) [11] found that extracts of MCVAS grown in Taiwan possessed more antioxidants than MC. MCVAS extracted using water (SC_50_ = 0.13 mg/mL) exhibited stronger DPPH scavenging activity than that extracted with ethanol (SC_50_ = 0.16 mg/mL).

The antioxidant capacity of plants may change under different storage conditions [21]. A study reported that partial vacuum storage resulted in slower deterioration of primed bitter gourd seeds compared with nonvacuum storage [22]. Storage temperature determines the rate of quality deterioration and the shelf life of frozen vegetables [23]. Improper storage causes changes in nutritional value [23]. The antioxidant capacity of cut or whole bitter gourds was lower when stored at 10 °C than that when stored at 2 °C for 7 days [24]. This revealed that a low storage temperature can reduce the loss of antioxidant activity. In frozen storage at −18 °C, the total phenolic content (TPC) of unblanched and blanched bitter gourds underwent little change for 90 days before gradually declining thereafter. However, at −40 °C, they remained nearly unchanged for 180 days [23].

Some studies have discussed differences in the antioxidant activities of various types of bitter gourds and between different parts of the bitter gourd, including the leaf, stem, and fruit. However, few studies have reported differences in the antioxidant properties of MC and MCVAS during storage. Bitter gourd is customarily used as a vegetable for direct cooking [6]. Dried slices and powder form are also applied [25]. The juice is another form of bitter gourd used for food [18]. The antioxidant activities of MC and MCVAS juice solutions were less studied in the literature. Few studies have reported differences in the antioxidant properties of MC and MCVAS juice solutions during storage. Therefore, the purpose of this study aimed to analyze the effects of storage time and temperature on the DPPH scavenging ability, ferric reducing power (FRP), and the TPC of MC and MCVAS juice solutions to have best applications in the future.

## 2. Materials and Methods

### 2.1. Reagent

**Figure 1:**
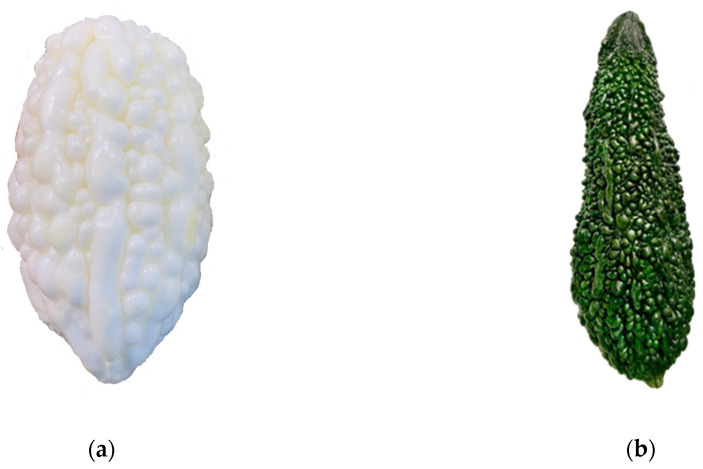
Bitter gourds used in this study. (**a**) *Momordica charantia* L., (**b**) *Momordica charantia* L. var. *abbreviata* Ser.

MC and MCVAS (Figure 1) were obtained from a local market in Taichung, Taiwan. Methanol was obtained from Aencore Chemical Co. (Surrey Hills, Australia). DPPH was purchased from Sigma Chemical Co. (St. Louis, MO, USA). Sodium carbonate (Na_2_CO_3_), trichloroacetic acid (C_2_HCl_3_O_2_), and sodium phosphate dibasic dihydrate (Na_2_HPO_4_·2H_2_O) were obtained from Riedel-de Haën Chemical Co. (Seelze, Germany). Sodium phosphate monobasic (NaH_2_PO_4_·H_2_O) was purchased from Shimakyu's Pure Chemicals Co. (Osaka, Japan). Iron(III) chloride hexahydrate (FeCl_3_·6H_2_O) was obtained from J. T. Baker Chemical Co. (Phillipsburg, NJ, USA). Potassium ferricyanide (K_3_Fe(CN)_6_) was obtained from First Chemical Co. (Pascagoula, MS, USA). Gallic acid was obtained from Fluka (Neu-Ulm, Germany). Folin–Ciocalteu reagent was obtained from Fisher Scientific Ltd. (Loughborough, Leicestershire, UK).

### 2.2. Sample Preparation

The fresh fruits of MC and MCVAS were washed and dried in airy shade. Then, the fruits were cut in half lengthwise, and the seeds were removed. The flesh was then cut into small pieces, and fresh juice was prepared using a Vitamix TNC5200 juicer (Vitamix Corporation, Cleveland, OH, USA). The juice was centrifuged at 2616× g for 5 min at room temperature. From the 2288.94 g of the sliced fruit of the MC, the weight of the clear supernatant was 1661.58 g. From the 2479.34 g of the sliced fruit of the MCVAS, the weight of the clear supernatant was 1704.51 g. Therefore, the average yields of MC and MCVAS juice were approximately 72.59% and 68.75%, respectively. In addition, fresh juice was freeze-dried to obtain the dry weight (DW) of the juice components and determine its concentration in fresh juice. The concentrations of the fresh MC and MCVAS juice were 15.60 and 16.51 mg/mL, respectively. The fresh MC and MCVAS juice samples were stored at −30 °C until assays without further processing except for dilution. The MC and MCVAS juice solution with a known lower concentration was obtained by dilution of the fresh juice.

### 2.3. Storage Conditions

The MC and MCVAS juice samples were maintained at 4, 25, and 37 °C for 24 days, and benzoic acid (0.56 g/kg) was added to the samples for preservation. The samples were examined directly at different storage times. All experiments were performed at least 6 times, and the evaluated data were presented as the mean ± standard error.

### 2.4. Liquid Chromatography–Mass Spectrometry Analysis

Ultrafast liquid chromatography (UFLC; UFLC-20ADXR) with a triple quadrupole mass spectrometer (LCMS-2020; Shimadzu, Kyoto, Japan) and a Shim-pack XR-ODS II column (2.2 μm, 2 mm × 100 mm, Shimadzu) was used for analysis. The mobile phase comprised a mixture of mobile phases A (0.1% formic acid and 1 g/L ammonium acetate in water) and B (0.1% formic acid and 1 g/L ammonium acetate in methanol) with a ratio of 100–70% in A in 0–40 min; 70–0% in A and 30–100% at 40–70 min; 0–100% in A at 70–70.1 min, and 100% in A at 70.1–80 min. The mobile phase flow rate was 0.4 mL/min, and the column temperature was maintained at 40 °C. UFLC–mass spectrometry (MS) used positive and negative electrospray ionization sources. MS detection was arranged as a full scan range from 100 to 1200 amu and operated at an interface voltage of 4.5 kV with a desolvation temperature of 150 °C. In addition, the flow rates of nebulizing and drying gas with nitrogen were 1.5 and 20 L/min, respectively [26].

### 2.5. 1,1-Diphenyl-2-Picrylhydrazyl Scavenging Ability Assay

DPPH scavenging activity was measured spectrophotometrically according to the method reported in previous papers [27,28,29,30,31,32]. The bitter gourd sample (2 mL juice sample) and the 0.08 mg/mL DPPH solution prepared with methanol (2 mL) were mixed together. Then, the mixture was shaken and incubated for 30 min in the dark at room temperature. Absorbance was measured at 517 nm, and DPPH scavenging activity was calculated using the following equation:

(1) $$\mathrm{DPPH}\;\mathrm{scavenging}\;\mathrm{ability}\;(\%)=\left[1-\left(\frac{A_{Sample}}{A_{Blank}}\right)\right]\times 100\%$$

### 2.6. Ferric Reducing Power Assay

The reducing power of the extracts was monitored according to the procedure described previously [27,30,32]. A 1-mL juice sample was mixed with 1 mL of 1% K_3_Fe(CN)_6_ solution and 1 mL of phosphate buffer (2 mM, pH 6.6), which comprised Na_2_HPO_4_·2H_2_O and NaH_2_PO_4_·H_2_O. After incubation for 20 min at 50 °C, 100 μL of 10% C_2_HCl_3_O_2_, 1.5 mL of distilled water, and 0.3 mL of 0.1% FeCl_3_·6H_2_O were added to the mixture for 10 min. The absorbance was measured at 700 nm. FRP was expressed as the milligram ascorbic acid equivalent (AAE) per gram of juice DW.

### 2.7. Determination of Total Phenolic Content

TPC was determined according to the Folin–Ciocalteu colorimetric assay following the method described in previous reports [28,29,30,31,32,33]. The bitter gourd sample (1 mL juice sample) was added to 1 mL of 1N Folin–Ciocalteu reagent and 0.8 mL of 7.5% Na_2_CO_3_ solution. Then, the mixture was shaken and kept in the dark at room temperature for 30 min and centrifuged at 2616× g for 5 min. Absorbance was measured at 765 nm with a spectrophotometer. TPC was expressed as milligram gallic acid equivalent (GAE) per gram of juice DW.

## 3. Results and Discussion

### 3.1. Liquid Chromatography–Mass Spectrometry Analysis

**Figure 2:**
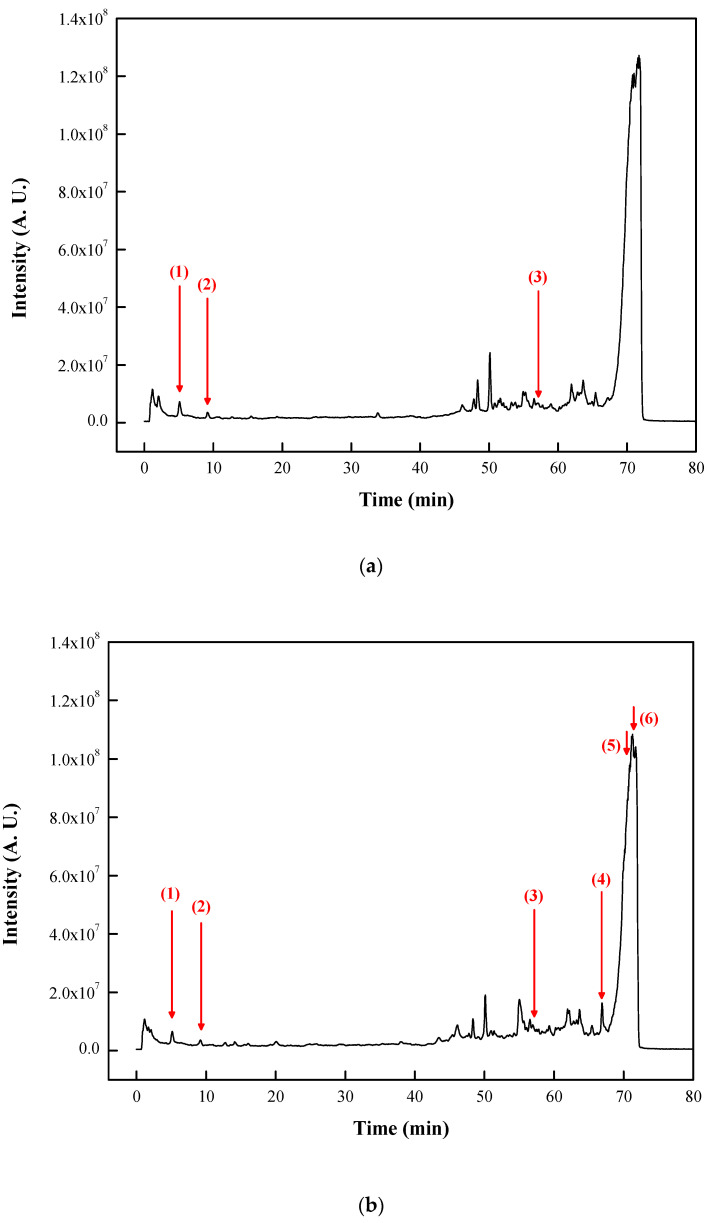
Liquid chromatography–mass spectrometry results of (**a**) *Momordica charantia* L. and (**b**) *Momordica charantia* L. var. *abbreviata* Ser. juice. Identified components included (1) phenylalanine; (2) tryptophan; (3) balsaminoside C; (4) goyaglycoside G; (5) vicine; and (6) momordicoside Q.

Figure 2 presents the LC–MS results of the MC and MCVAS juices. This study compared their molecular weights with those of previous studies and identified (1) phenylalanine [34], (2) tryptophan [35], and (3) balsaminoside C [36] as the major components of MC juice. Moreover, (1) phenylalanine, (2) tryptophan, (3) balsaminoside C, (4) goyaglycoside G [37], (5) vicine [38], and (6) momordicoside Q [36] were identified as the major components of MCVAS juice. Table 1 presents the mass data of these compounds in the two juice solutions.

Some biological activities, including antioxidant activity of these identified compounds in the juice in Table 1, have been reported. For example, tryptophan is a well-known antioxidant against peroxyl radicals and can exert its antioxidant ability by electron transfer mechanism or hydrogen atom transfer [39]. Phenylalanine with phenolic hydroxyl group has DPPH scavenging activity and ferric ion reducing power [40]. Balsaminoside C is a cucurbitane-type triterpenoid and can serve as an antioxidant agent for the treatment of reactive oxygen species-induced diseases [41].

### 3.2. Effects of Concentration on Antioxidant Activity

**Figure 3:**
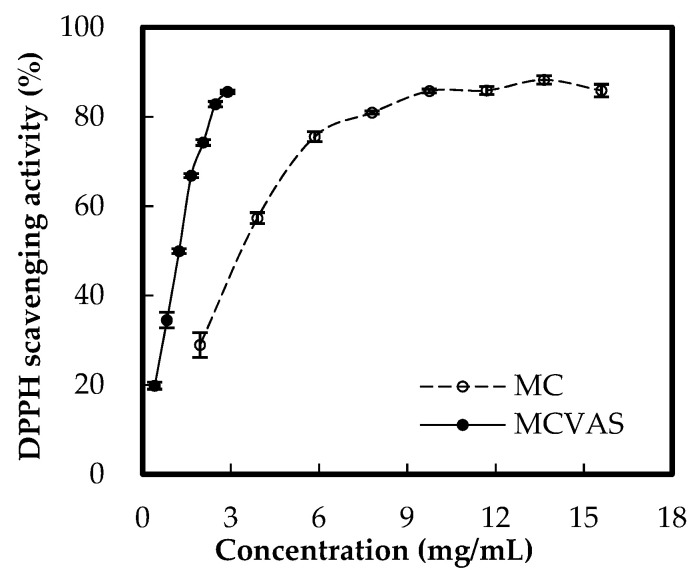
Effects of sample concentration on 1,1-diphenyl-2-picrylhydrazyl scavenging activity.

Figure 3 illustrates that the antioxidant activity in terms of DPPH scavenging ability increased with the concentrations of MC and MCVAS. Moreover, the DPPH scavenging activity of MCVAS was higher than that of MC. The SC_50_ values of ascorbic acid and Trolox, well-known antioxidant standards, are 8.35 μg/mL [32] and 10.51 μg/mL [42], respectively. According to Figure 3, the SC_50_ values were 3.33 ± 0.10 and 1.19 ± 0.03 mg/mL for MC and MCVAS, respectively. The DPPH SC_50_ value of MCVAS was lower than that of MC, which indicated that the DPPH scavenging ability of MCVAS was stronger than that of MC. According to literature reports [7,17], the DPPH SC_50_ values of MC extracts vary with the extraction method; however, our result on MCVAS is comparable with those obtained by Krishnendu and Nandini [7].

**Figure 4:**
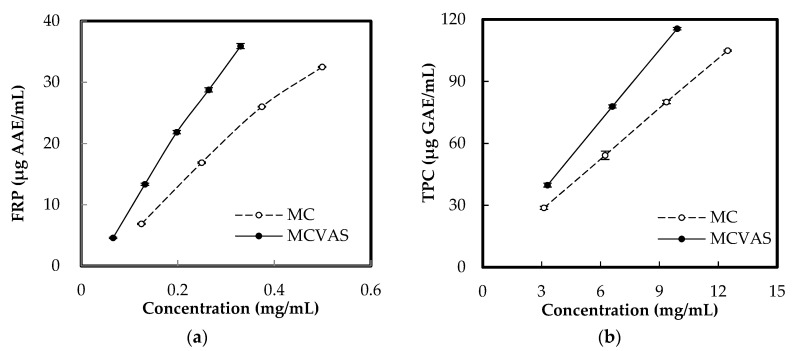
Effects of sample concentration on (**a**) ferric reducing power and (**b**) total phenolic content.

Figure 4 illustrates the effects of the concentration on FRP and TPC in MC and MCVAS. As observed in Figure 4a, the FRP of MC and MCVAS increased with concentration. The FRP of MCVAS was higher than that of MC, and the effect of concentration on FRP for MCVAS was stronger than that for MC. Through regression analysis, the FRP values were determined to be 68.93 ± 3.32 and 118.14 ± 17.60 mg of AAE/g DW for MC and MCVAS, respectively. This corresponded to the reported FRP value of 10 ± 0.4 mg of GAE/g for bitter gourd [43] (approximately 87.02 mg of AAE/g by conversion [44]). Figure 4b shows the values of the TPC increasing with rising concentration and the nearly linear relationship between TPC and concentration. Moreover, the TPC of MCVAS was higher than that of MC, and the effect of concentration on TPC was more conspicuous for MCVAS than for MC. Through regression analysis, the TPC values were determined to be 8.15 ± 0.51 and 11.47 ± 0.49 mg of GAE/g DW for MC and MCVAS, respectively. Table 2 summarizes the FRP and TPC values of MC and MCVAS in this study for comparison.

The various extraction conditions of MC and MCVAS may have affected TPC and resulted in a wide range. According to literature reports, the TPC values ranged from 0.54 to 135.33 mg of GAE/g DW [45,46]. The determined TPC of the MC and MCVAS juice in this study fell within the range. Agreeing with a previous study [11], MCVAS possessed higher TPC content than MC. A previous study reported that the antioxidant activity of bitter gourd extracts was related to the presence of phenolic compounds [17]. Seven phenolic compounds were identified, namely *p*-coumaric acid, tannic acid, benzoic acid, ferulic acid, gallic acid, caffeic acid, and (+)-catechin, and the predominant phenolic compounds were gallic acid, followed by caffeic acid and catechin [3,6,17].

### 3.3. Effect of Storage Time and Storage Temperature

#### 3.3.1. Solution Appearance

A 24-d observance was conducted on the MC and MCVAS juice. The results indicated that MC and MCVAS juice kept at 4 and 25 °C appeared clear after 24-d storage. However, the juice of MC and MCVAS kept at 37 °C became moldy after 12 days, even though benzoic acid was added for preservation. Therefore, the antioxidant activity was not determined for juice kept at 37 °C after 12 days.

#### 3.3.2. 1,1-Diphenyl-2-picrylhydrazyl Scavenging Activity

**Figure 5:**
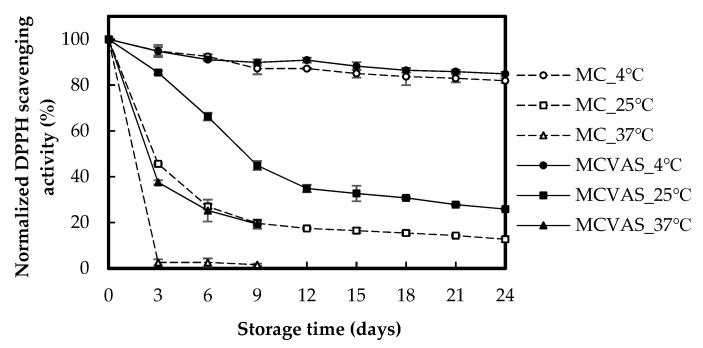
Effects of storage time on the normalized 1,1-diphenyl-2-picrylhydrazyl scavenging activity of *Momordica charantia* L. and *Momordica charantia* L. var. *abbreviata* Ser.

Figure 5 presents the DPPH scavenging activities of MC and MCVAS kept at varying temperatures for different storage times. The normalized DPPH scavenging ability of MC decreased from 100% to 81.90% when kept at 4 °C for 24 days, to 12.71% when kept at 25 °C for 24 days, and to 1.62% when kept at 37 °C for 9 days. After 3 days, the normalized DPPH scavenging ability of MC had decreased by 97.41% when stored at 37 °C and by 54.36% when stored at 25 °C. However, only a 5.15% decrease was noted for that stored at 4 °C. The normalized DPPH scavenging ability of MCVAS decreased from 100% to 84.87% when kept at 4 °C for 24 days, 25.47% when kept at 25 °C for 24 days, and 19.33% when kept at 37 °C for 9 days. Substantial changes in the DPPH scavenging activity of MCVAS occurred on the third day, and the activity decreased by 62.45% when kept at 37 °C. However, only 5.23% and 14.30% decreases were observed for that kept at 4 °C and 25 °C, respectively. Therefore, the DPPH scavenging abilities of MC and MCVAS decreased with storage time, and the decrease depended on the storage temperature. The loss of DPPH scavenging ability in MC with storage time was greater than that of MCVAS. The effect of storage time on DPPH scavenging ability was more prominent at higher storage temperatures, indicating that lower storage temperature could mitigate the decreasing DPPH scavenging activity. This result was similar to that reported by Myojin et al. [23], who indicated that DPPH scavenging activity decreased more at higher storage temperatures.

#### 3.3.3. Ferric Reducing Power

**Figure 6:**
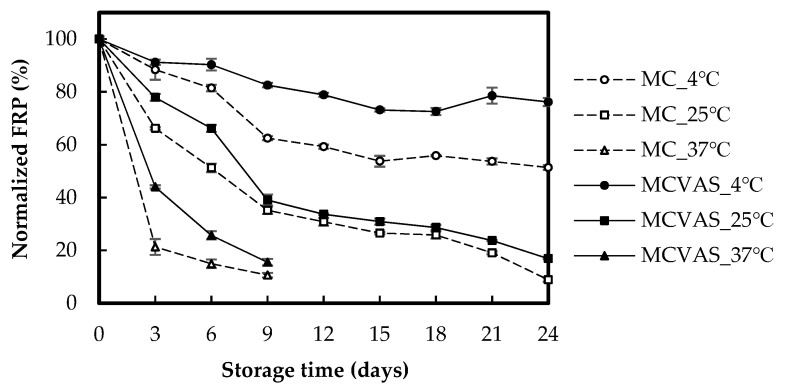
Effects of storage time on the normalized ferric reducing power of *Momordica charantia* L. and *Momordica charantia* L. var. *abbreviata* Ser.

Figure 6 presents the FRP of MC and MCVAS under different storage times and storage temperatures. The value of normalized FRP for MC fell from 100% to 51.36% when kept at 4 °C for 24 days, to 8.86% when kept at 25 °C for 24 days, and to 10.62% when kept at 37 °C for 9 days. Moreover, a rapid decrease was observed on the third day, and MC kept at 37 °C had the largest decline in normalized FRP (78.70%). The normalized FRP of MC kept at 4 °C and 25 °C had decreased by 11.61% and 33.81% on the third day, respectively. The normalized FRP value for MCVAS declined from 100% to 76.15% when kept at 4 °C for 24 days, to 16.85% when kept at 25 °C for 24 days, and to 15.48% when kept at 37 °C for 9 days. On the third day, the normalized FRP values of MCVAS kept at 4 °C, 25 °C and 37 °C had decreased by 8.79%, 22.10%, and 55.98%, respectively. Therefore, a rapid decrease in normalized FRP occurred when kept at 37 °C. Similar to the results for DPPH scavenging activity, the decline in normalized FRP with storage time was more apparent for MC than for MCVAS. The results indicated that keeping the juice of MC and MCVAS at higher storage temperatures accelerated the decrease in normalized FRP.

#### 3.3.4. Total Phenolic Content

**Figure 7:**
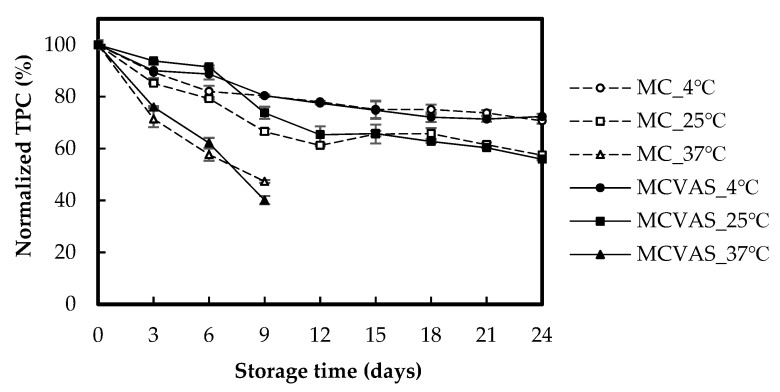
Effects of storage time on the normalized total phenolic content of *Momordica charantia* L. and *Momordica charantia* L. var. *abbreviata* Ser.

Figure 7 depicts the effects of storage time and temperature on TPC for MC and MCVAS. The normalized TPC for MC declined from 100% to 70.69% when kept at 4 °C for 24 days, to 57.46% when kept at 25 °C for 24 days, and to 47.35% when kept at 37 °C for 9 days. The normalized TPC for MCVAS decreased from 100% to 72.36% when kept at 4 °C for 24 days, to 55.87% when kept at 25 °C for 24 days, and to 40.11% when kept at 37 °C for 9 days. As shown in Figure 7, The TPC values of MC and MCVAS kept at 37 °C declined rapidly within 9 days, with declines of 52.65% and 59.89% observed in MC and MCVAS, respectively. Over 9 days of storage, the TPC values of MC and MCVAS decreased by 19.63% and 19.60%, respectively, when kept at 4 °C and by 33.46% and 26.24%, respectively, when kept at 25 °C. Overall, no clear difference between MC and MCVAS appeared during the 24-day storage. Compared with 25 °C and 37 °C, 4 °C was the optimal storage temperature. Higher storage temperature resulted in more decline in TPC, which was in agreement with the findings of a previous report [23].

The dynamic decay of DPPH radical scavenging activity (Figure 5) and TPC (Figure 7) of the juice is not similar. Therefore, DPPH radical scavenging activity is little related to TPC of the juice. The inconsistency between DPPH radical scavenging activity and TPC is also found in previous studies for MC [18], *Pleurotus porrigens* extracts [47], and selected phenolic acids [48].

Overall, storage has great influence on antioxidant activities of juice from MC and MCVAS. The identified compounds in the juice may change in the storage process and affect the antioxidant activities of juice. For example, there is a loss in tryptophan during storage [49], and the antioxidant activity decays with time.

## 4. Conclusions

This study investigated the antioxidant activities of MC and MCVAS juice and explored the effects of storage time and temperature on their antioxidant activities. The antioxidant activity analysis included DPPH scavenging activity, FRP, and TPC. The results indicated that MCVAS had significantly higher antioxidant activity than MC. Overall, the antioxidant activity of MC and MCVAS decreased substantially after 3 days of storage, and antioxidant activity decreased more rapidly at higher storage temperatures. A lower temperature could alleviate the decrease in the antioxidant activity of MC and MCVAS juice. 

## Figures and Tables

**Table 1:** Mass data of identified compounds in *Momordica charantia* L. and *Momordica charantia* L. var. *abbreviata* Ser. juice solutions.

No.	Molecular Weight (g/mol)	Retention Time (min)	(+)ESI (*m/z*)	(−)ESI (*m/z*)
1	165.19	5.2	166.05 [M + H]^+^	-
2	204.23	9.1	205.05 [M + H]^+^	-
3	620.87	57.1	-	619.30 [M − H]^−^
4	811.02	67.0	812.50 [M + H]^+^	-
5	304.26	70.0	327.20 [M + Na]^+^	-
6	652.87	71.1	675.55 [M + Na]^+^	-

**Table 2:** Ferric reducing power and total phenolic content values of *Momordica charantia* L. and *Momordica charantia* L. var. *abbreviata* Ser. in this study.

Properties	Species	
	MC	MCVAS
FRP (mg AAE/g DW)	68.93 ± 3.32	118.14 ± 17.60
TPC (mg GAE/g DW)	8.15 ± 0.51	11.47 ± 0.49
